# Oral treatment with a zinc complex of acetylsalicylic acid prevents diabetic cardiomyopathy in a rat model of type-2 diabetes: activation of the Akt pathway

**DOI:** 10.1186/s12933-016-0383-8

**Published:** 2016-05-06

**Authors:** Sevil Korkmaz-Icöz, Samer Al Said, Tamás Radovits, Shiliang Li, Maik Brune, Péter Hegedűs, Ayhan Atmanli, Mihály Ruppert, Paige Brlecic, Lorenz Heyne Lehmann, Bernd Lahrmann, Niels Grabe, Yutaka Yoshikawa, Hiroyuki Yasui, Patrick Most, Matthias Karck, Gábor Szabó

**Affiliations:** Laboratory of Cardiac Surgery, Department of Cardiac Surgery, University Hospital Heidelberg, Im Neuenheimer Feld 326, 69120 Heidelberg, Germany; Heart and Vascular Center, Semmelweis University, Városmajor u. 68, Budapest, 1122 Hungary; Department of Internal Medicine I and Clinical Chemistry, University Hospital Heidelberg, Im Neuenheimer Feld 671, 69120 Heidelberg, Germany; Department of Cardiology, Angiology and Pulmonology, University Hospital Heidelberg, Im Neuenheimer Feld 410, 69120 Heidelberg, Germany; Hamamatsu Tissue Imaging and Analysis Center (TIGA), Bioquant, University of Heidelberg, 69120 Heidelberg, Germany; Steinbeis Transfer Center for Medical Systems Biology, 69124 Heidelberg, Germany; Department of Medical Oncology, National Center for Tumor Diseases, University of Heidelberg, 69120 Heidelberg, Germany; Department of Analytical and Bioinorganic Chemistry, Kyoto Pharmaceutical University, Kyoto, 607-8414 Japan; Molecular and Translational Cardiology, Department of Internal Medicine III, University Hospital Heidelberg, Im Neuenheimer Feld 410, 69120, Heidelberg Germany

**Keywords:** Cardiac function, Diabetic cardiomyopathy, Type-2 diabetes mellitus, Zinc-aspirin complex

## Abstract

**Background:**

Type-2 diabetics have an increased risk of cardiomyopathy, and heart failure is a major cause of death among these patients. Growing evidence indicates that proinflammatory cytokines may induce the development of insulin resistance, and that anti-inflammatory medications may reverse this process. We investigated the effects of the oral administration of zinc and acetylsalicylic acid, in the form of bis(aspirinato)zinc(II)-complex Zn(ASA)_2_, on different aspects of cardiac damage in Zucker diabetic fatty (ZDF) rats, an experimental model of type-2 diabetic cardiomyopathy.

**Methods:**

Nondiabetic control (ZL) and ZDF rats were treated orally with vehicle or Zn(ASA)_2_ for 24 days. At the age of 29–30 weeks, the electrical activities, left-ventricular functional parameters and left-ventricular wall thicknesses were assessed. Nitrotyrosine immunohistochemistry, TUNEL-assay, and hematoxylin-eosin staining were performed. The protein expression of the insulin-receptor and PI3K/AKT pathway were quantified by Western blot.

**Results:**

Zn(ASA)_2_-treatment significantly decreased plasma glucose concentration in ZDF rats (39.0 ± 3.6 vs 49.4 ± 2.8 mM, P < 0.05) while serum insulin-levels were similar among the groups. Data from cardiac catheterization showed that Zn(ASA)_2_ normalized the increased left-ventricular diastolic stiffness (end-diastolic pressure–volume relationship: 0.064 ± 0.008 vs 0.084 ± 0.014 mmHg/µl; end-diastolic pressure: 6.5 ± 0.6 vs 7.9 ± 0.7 mmHg, P < 0.05). Furthermore, ECG-recordings revealed a restoration of prolonged QT-intervals (63 ± 3 vs 83 ± 4 ms, P < 0.05) with Zn(ASA)_2_. Left-ventricular wall thickness, assessed by echocardiography, did not differ among the groups. However histological examination revealed an increase in the cardiomyocytes’ transverse cross-section area in ZDF compared to the ZL rats, which was significantly decreased after Zn(ASA)_2_-treatment. Additionally, a significant fibrotic remodeling was observed in the diabetic rats compared to ZL rats, and Zn(ASA)_2_-administered ZDF rats showed a similar collagen content as ZL animals. In diabetic hearts Zn(ASA)_2_ significantly decreased DNA-fragmentation, and nitro-oxidative stress, and up-regulated myocardial phosphorylated-AKT/AKT protein expression. Zn(ASA)_2_ reduced cardiomyocyte death in a cellular model of oxidative stress. Zn(ASA)_2_ had no effects on altered myocardial CD36, GLUT-4, and PI3K protein expression.

**Conclusions:**

We demonstrated that treatment of type-2 diabetic rats with Zn(ASA)_2_ reduced plasma glucose-levels and prevented diabetic cardiomyopathy. The increased myocardial AKT activation could, in part, help to explain the cardioprotective effects of Zn(ASA)_2_. The oral administration of Zn(ASA)_2_ may have therapeutic potential, aiming to prevent/treat cardiac complications in type-2 diabetic patients.

## Background

Diabetes mellitus (DM) is a metabolic disorder characterized by chronic hyperglycemia due to an impairment of insulin secretion, defects of insulin action, or both. The increasing prevalence of DM, and its association with cardiovascular disease have become serious public health issues. Primarily, the complications of diabetes have been attributed to increased atherosclerosis. However, DM is also able to alter the cardiac structure and function in the absence of coronary atherosclerosis, hypertension and significant valvular disease [1], a condition called diabetic cardiomyopathy (DCM) [2, 3]. Depending on its cause, diabetes can be classified into two main categories, type-1 and type-2 DM. More than 90 % of diabetic patients are diagnosed with type-2 diabetes dominated by hyperinsulinemia, hyperglycemia and dyslipidemia. Additionally, about 80 % of type-2 diabetic patients are overweight or obese. Importantly, obesity is not only associated with lipid accumulation in adipose tissue, but also in non-adipose tissues such as the myocardium. This lipid overload in the cytoplasm of the cells may cause cellular dysfunction, cell death, eventual cardiac dysfunction and heart failure, and also may be an explanation for DCM [4–6]. Additionally, the direct effects of hyperglycemia on cardiomyocytes and oxidative stress due to the production of reactive oxygen species and nitrogen species, have also been proposed to be the root cause underlying the development of DCM [7]. Furthermore, recent studies have revealed that proinflammatory cytokines can cause the sustained development of insulin resistance [8], and that anti-inflammatory medications may reverse this process [9, 10]. Thus therapeutic interventions targeting free radical generation, inflammation, and/or endogenous antioxidant enzymes enhancement, and glycemic metabolism by using insulin-enhancing or insulin-mimetic agents would be useful in patients with type-2 DM at risk for developing DCM.

In recent years some metal ions and their complexes have been proposed as candidates for treating DM. Zinc is an essential trace element for the synthesis, storage, and release of insulin [11]. Orally active zinc(II)-complexes have been found to have in vitro insulinomimetic activity and an in vivo blood glucose lowering effect [12]. In addition to glycemic control, zinc(II) ions are known to possess anti-ulcer activities as well as anti-inflammatory properties [13]. Therefore, the dietary supplementation of zinc has been recommended as a novel strategy to manage diabetes-associated cardiac complications [14]. The proposed molecular mechanism of zinc consists of the activation of the entire insulin signalling pathway, including the activation of protein kinase B (also known as AKT), a serine/threonine-specific protein kinase. Thus, lipogenesis is increased and the glucose transport (GLUT)-4 protein is translocated to the cell membrane, facilitating the uptake of glucose into the cell [11]. Moreover, antioxidant activities of zinc complexes have been described [15–17], implying the activation of antioxidant enzymes, the attenuation of oxygen-derived free radicals, and the generation of free radicals scavengers. A high dose of salicylates has been found to reverse hyperglycemia and hyperinsulinemia in type-2 diabetic patients [10]. Therefore, the zinc complex of acetylsalicylic acid (aspirin) (Zn(ASA)_2_) was synthesized [18]. The anti-inflammatory effect of aspirin can be increased, the gastrointestinal absorption of aspirin may be enhanced, the ulcergenicity of aspirin may be reduced, and zinc can be presented in a better tolerated dosage form. Recently, it has been shown that the synergic effect of zinc and aspirin, in the form of bis(aspirinato)zinc(II) complex improved not only hyperglycemia, insulin resistance, leptin resistance, and hypoadiponectinemia, but also improved hypertension in type-2 diabetic KK-Ay mice [19]. However, experimental investigations of the effects of the zinc complex of acetylsalicylic acid on diabetic hearts using in vivo animal models are yet lacking.

The Zucker diabetic fatty (ZDF) rat is the most widely used type-2 diabetic obese rodent model. At the age of 25 weeks ZDF rats develop a severe metabolic syndrome associated with profound modifications of the cardiac transcriptome, which may be involved in the development of cardiac pathology [20]. Additionally, old ZDF rats show gender-related differences, such as female rodents are less susceptible to high-fat diet-induced insulin resistance, that might be relevant to humans [21].

One of the therapeutic options concerning diabetic patients without cardiovascular disease or with ischemic heart disease, includes aspirin treatment. We hypothesized that the use of zinc and aspirin in the form of bis(aspirinato)Zinc(II) complex, may be a new treatment concept for these patients due to its blood glucose lowering function [19], cardioprotective actions [22], and preventive effects against restenosis [23]. In light of these findings, we investigated the effects of an oral administration of the zinc complex of acetylsalicylic acid upon diabetes-induced changes in cardiac structure, function, and electrical activity. As the AKT-GLUT-4 signalling pathway has been found to be activated by zinc complexes, we also investigated whether their protein expressions play a crucial role in our experimental setting.

## Methods

### Animals

Male Zucker diabetic fatty (ZDF) rats and Zucker lean (ZL) control rats were obtained at an age of 12 weeks (Charles River, Saint Germain-Nuelles, France). The Animals were kept in local facilities, housed in standard cages with 12 h light/dark cycles at a constant temperature of 22 ± 2 °C, a Purina 5008 diet, as recommended by the supplier, was fed with water ad libitum. Both the care of the animals and the experimental procedures were conducted according to the ‘Principles of Laboratory Animal Care’ drafted by the National Society for Medical Research and the ‘Guide for the Care and Use of Laboratory Animals’, prepared by the Institute of Laboratory Animal Resources and published by the National Institute of Health (NIH Publication No. 86-23, revised 1996). The experiments were approved by the Ethical Committee of the Land Baden-Württemberg for Animal Experimentation (G-56/13).

### Rat model of type-2 DM

The ZDF male rat (*fa*/*fa)* is genetically inbred and develops obesity, fasting hyperglycemia, and type-2 diabetes (ZDF group, n = 13) while fed a special diet. The homozygous (+/+) and heterozygous (*fa*/+) Zucker genotypes remain lean (ZL group, n = 20). Based on literature data and the results of our previous studies [24, 25], final experiments on the rats were performed at an age of 29–30 weeks.

### Experimental groups

**Fig. 1:**
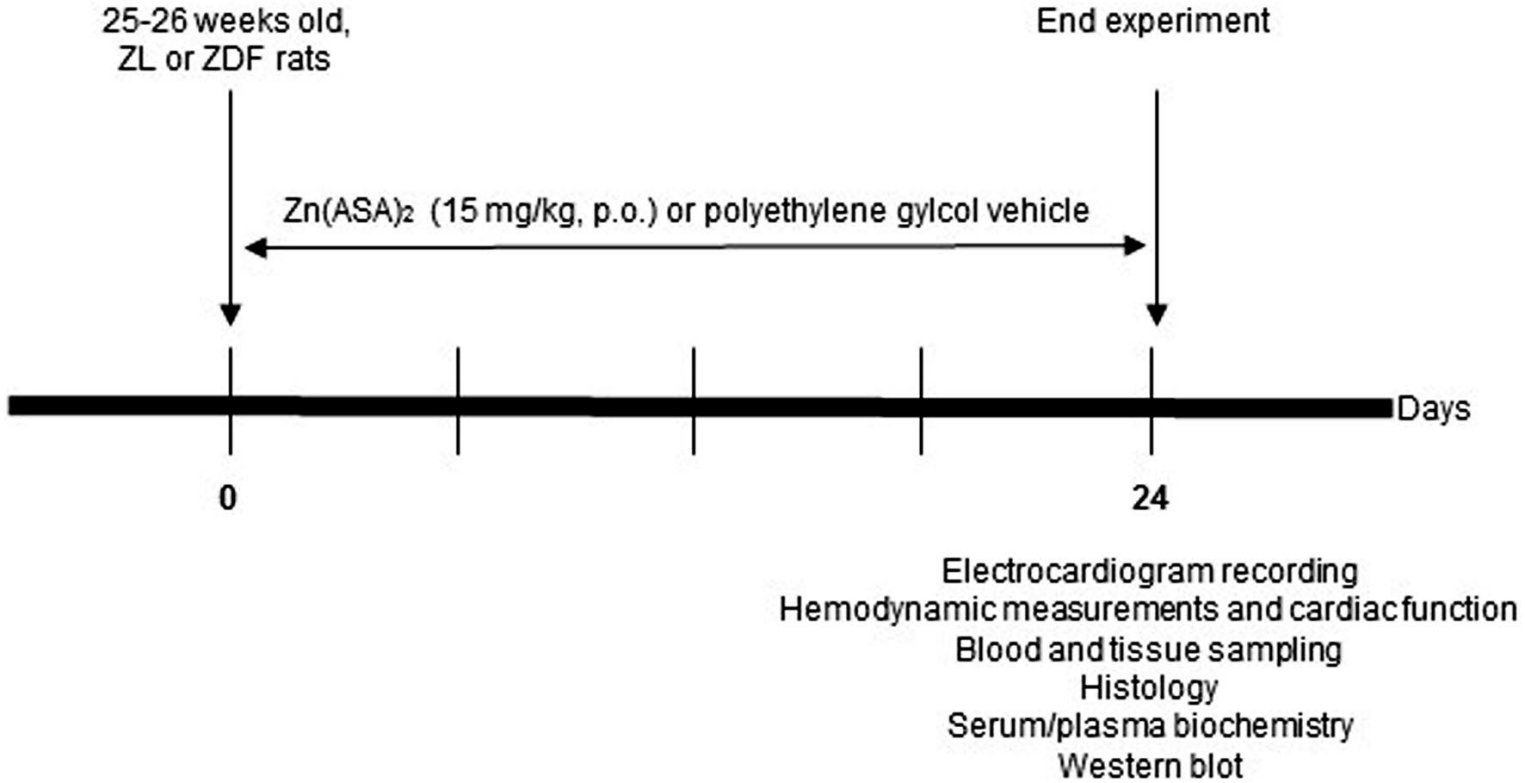
Experimental protocol. At the age of 25–26 week-old, ZL or ZDF rats were treated with Zn(ASA)_2_ or a polyethylene glycol vehicle (15 mg/kg) orally for 24 consecutive days. *Zn(ASA)*_2_ indicates a zinc complex of acetylsalicylic acid, *ZDF* Zucker diabetic fatty rats, *ZL* Zucker lean

At the age of 25–26 week-old the rats were randomly divided into four groups: (1) ZL group (n = 10): ZL rats received a polyethylene glycol vehicle, (2) ZDF group (n = 7): ZDF rats received a polyethylene glycol vehicle, (3) ZDF + Zn(ASA)_2_ group (n = 6): ZDF rats were treated with Zn(ASA)_2_, and (4) ZL + Zn(ASA)_2_ group (n = 10): ZL rats received Zn(ASA)_2_. Zn(ASA)_2_ was prepared as a suspension in a polyethylene glycol vehicle at a volume of 2 ml/kg. It was administered once daily, by oral gavage, for 24 days at the dose of 15 mg/kg. The application and dosage of Zn(ASA)_2_ were determined according to the results of previous rodent experiments [19]. The experimental protocol is shown in Fig. 1.

### Echocardiography

One day before the final experiments, the rats were slightly anesthetized with 1.5–2.0 % isoflurane by mask, the left side of the chest was shaved to obtain a clear image, and the animals were situated in the supine position on a warming pad. Two-dimensional (long- and short-axis) and motion-mode (M-mode) images of the left ventricle were obtained from a parasternal view, carried out with vevo 2100 ultrasound system, and using standard echocardiography. All data was analysed off-line at the end of the study, with the software resident on the ultrasound system. Interventricular diameters including left ventricular internal end-diastolic dimensions (LVIDd) and left ventricular internal end-systolic dimensions (LVIDs), were measured. All measurements were based on the mean of at least three consecutive cardiac cycles on the M-mode tracing at the level of the papillary muscle, and the mean values were used in our analyses.

### Electrocardiogram (ECG) recording

The rats were anesthetized with sodium pentobarbital (60 mg/kg) intraperitoneally, and they were kept in a supine position. Their body temperature (measured via rectal probe) was maintained at 37 °C. The standard 12-lead electrocardiogram (leads I, II, III, aVR, aVL, aVF, V1, V2, V3, V4, V5, and V6) was recorded using subcutaneously placed needle electrodes. All leads were connected to a standard direct-writing recorder (Mortara Instrument, WI, USA). The paper speed was set at 50 mm/s, and the sensitivity at 10 mm/mV. The ECG analysis was evaluated by lead II including the following measurements: ST-segment elevation, QRS complex, and QT-intervals. The QT-interval, measured from the onset of the QRS complex to the end of the T wave, was corrected using the normalized Bazett’s formula adjusted for rats (nQTc = QT/(RR/f)^1/2^) (RR corresponding to cardiac cycle length and f to frequency) [26]. The electrocardiography was analyzed by an examiner blinded to the experimental groups.

### In vivo hemodynamic measurements and cardiac function

After the ECG recording, the animals were tracheotomised, intubated, and artificially ventilated. A polyethylene catheter was inserted into the left external jugular vein for fluid administration purposes. A 2F microtip pressure–volume catheter (SPR-838, Millar Instruments, Houston, TX, USA) was inserted into the right carotid artery and advanced into the ascending aorta. After allowing an initial stability period of 5 min, the systolic blood pressure (SBP), diastolic blood pressure (DBP), mean arterial pressure (MAP), and heart rate were recorded. After that, the catheter was advanced into the left ventricle under pressure control. With the use of a special pressure–volume analysis program (PVAN, Millar Instruments, Houston, TX, USA), stroke volume, cardiac output (CO), stroke work, left-ventricular end-systolic pressure (LVESP), left-ventricular end-diastolic pressure (LVEDP), and time constant of left-ventricular (LV) pressure decay (Τau) were calculated. The pressure–volume relations of the LV cavity were assessed by briefly compressing the inferior vena cava. The slope [end-systolic elastance (E_es_)] of the LV end-systolic pressure–volume relationship (ESPVR) according to the parabolic curvilinear model [27] was calculated as a load-independent index of LV contractility. The slope of the LV end-diastolic pressure–volume relationship (EDPVR) was calculated as a reliable index of LV stiffness.

### Biochemical estimation

After the heart function was measured the blood was collected from the abdominal aorta in Lithium-Heparin-Gel Monovette^®^ and serum tubes. Then, after centrifugation, plasma and serum samples were obtained. The levels of plasma glucose, high-density lipoprotein (HDL) cholesterol, total cholesterol, triglyceride and aspartate aminotransferase (AST) were determined in the central laboratory of the Heidelberg University clinic on an ADVIA 2400 chemistry analyser (Siemens, Germany). The levels of serum insulin were measured via ELISA kit (Mercodia, Uppsala, Sweden) and non-esterified fatty acids were determined with a commercially available enzymatic and colorimetric assay kit (Wako Chemicals, Germany). Fast protein liquid chromatography (FPLC) was used for the analysis of lipoprotein profiles. Serum samples from each experimental group were pooled and separated by chromatography on an ÄKTApurifier FPLC system (GE Healthcare, Sweden). Briefly, 400 μl of pooled serum were separated using a Superose-6 size exclusion column, and the eluate was fractionated. Total cholesterol and triglyceride contents of the individual fractions were determined via commercially available kits (Randox Laboratories, UK and Human Diagnostica, Germany, respectively).

### Histopathological process

After the blood samples were collected pieces of myocardial tissue were fixed in a buffered paraformaldehyde solution (4 %), embedded in paraffin, and cut to 5-μm. All of the histological evaluations were conducted by an examiner blinded to the experimental groups.

#### Hematoxylin and eosin staining

Paraffin-embedded sections were placed on adhesive slides and stained with hematoxylin and eosin. Cardiomyocyte cross-sectional areas were calculated under a microscope using the Cell^A software (Olympus Soft Imaging Solutions GmbH, Germany).

#### Terminal deoxynucleotidyl transferase-mediated dUTP nick end-labeling (TUNEL)

TUNEL assay was performed according to the manufacturer’s instructions (Chemicon International, Temecula, CA, USA), to detect DNA strand breakages (free 3′-OH DNA ends). Briefly, the rehydrated sections were digested with 20 μg/ml DNase-free Proteinase K (Sigma-Aldrich, Germany) to retrieve antigenic epitopes, and endogenous peroxidases were blocked with 3 % hydrogen peroxide (H_2_O_2_). The free 3ʹ-OH termini of the DNA ends were labeled with a reaction mixture of terminal deoxynucleotidyl transferase (TdT) and digoxigenin-dUTP at 37 °C for 1 h (Chemicon International, Temecula, CA, USA). Incorporated digoxigenin-conjugated nucleotides were detected using a horseradish peroxidase conjugated anti-digoxigenin antibody and 3,3ʹ-diaminobenzidine. The sections were counterstained with Gill’s hematoxylin. Dehydrated sections were cleared in xylene, mounted with Permount (Fischer Scientific, Germany), and coverslips were applied. Based on the intensity and distribution of the labelling, a semi-quantitative histomorphological assessment was performed using conventional microscopy. For the assessment of TUNEL-labelled cells, the number of positive cell nuclei per microscopic examination field (with 200× magnification) was counted in four fields for each sample, averaged, and the mean was calculated for each experimental group.

#### Acid fuchsin orange G (AFOG)

An AFOG-stain was used to detect collagen fibers in the heart tissue. Histological sections were automatically imaged, using 20× magnification (resolution: 0.46 μm/pixel), with the Hamamatsu NanoZoomer 2.0-HT Scan System (Hamamatsu Photonics, Hamamatsu Japan). The slide scanner automatically detects the region of interest containing the tissue, and also automatically determines a valid focal plane for scanning. Image processing algorithms have been developed using VisiomorphDP version 4.5.1.324 (Visiopharm, Hoersholm, Denmark). Image processing was performed in several distinguished steps: (a) Automatic region of interest detection using thresholding methods in the RGB-Colorspace. As a result of this step, the tissue regions are separated from the background for further analysis; (b) Pixelwise classification of the whole slide by using a linear Bayesian Classifier [28] in RGB colorspace. After training the classifier by manually marking background areas within the tissue regions, the normal muscle tissue, as well as the fibrotic areas, the classifier was able to separate the processed area into one of these three subcategories. A second subsequent classifier was trained to detect folded tissue and artifacts; (c) As a post-processing step, areas that were too small (small tissue fragments, staining artifacts, dust particles) were removed by using morphological operations like opening and closing. The ratio of the fibrotic area in relation to the total tissue can be calculated using these output variables.

#### Immunohistochemical detection of nitrotyrosine

According to previously described methods [29] we performed immunohistochemical staining on heart tissue to detect nitrotyrosine, a marker for nitro-oxidative stress. Positive immunohistological staining was scored using semiquantitative analysis. The total score was then given based on the sum of the intensity and the area scores. The intensity was evaluated as follows: no staining (score 0), weak staining (score 1), moderate staining (score 2), and severe staining (score 3). The area score was evaluated (from 0 to 4) within score 0 representing no staining, score 1 (positive cells <10 %), score 2 (positive cells 11–50 %), score 3 (positive cells 51–80 %) and score 4 (positive cells >80 %).

### Western blotting

Proteins were extracted from the heart tissue in a 1x RIPA buffer (Melford, Ipswich, UK). The protein concentration was determined with Bradford assay. Total protein homogenates 20 μg/30 μl were denatured, separated on sodium dodecyl sulfate polyacrylamide electrophoresis gels, and transferred to a polyvinylidene fluoride membrane (Millipore, Darmstadt, Germany). The membrane was blocked with 2.5 % BSA in Tris- Buffered Saline Tween 20 for 1 h, before being incubated overnight at 4 °C with CD36 (1:1000, Cell Signalling Technology, Cambridge, UK), GLUT-4 (1:100, Santa Cruz, Biotechnology, Heidelberg, Germany), insulin receptor-β (1:1000, Cell Signalling Technology, Cambridge, UK), phosphorylated-PI3K (1:1000, Cell Signalling Technology, Cambridge, UK), PI3K (1:1000, Cell Signalling Technology, Cambridge, UK), AKT, or phosphorylated-AKT (1:500, Abcam, Cambridge, UK). After washing the blots to remove excessive primary antibody binding, the blots were incubated for 1 h with a horseradish peroxydase conjugated secondary antibody at room temperature. Glyceraldehyde-3-phosphate dehydrogenase (GAPDH), housekeeping protein, was used for loading control and protein normalization. The immunoreactive protein bands were developed using the Enhanced Chemiluminescence system (PerkinElmer, Rodgau-Juegesheim, Germany). The intensity of the immunoblot bands was detected with Chemismart 5100.

### Cellular model of hydrogen peroxide-induced cardiomyocytes oxidative stress

#### Isolation and culture of neonatal rat cardiomyocytes, measurement protocol

Neonatal cardiomyocytes were isolated from 1- to 2-day-old newborn rats (Charles River, Sulzfeld, Germany). Briefly, the hearts were excised and rapidly placed into an ice-cold buffer (in mmol/L: NaCl 116.4, HEPES 20, NaH_2_PO_4_ 1, glucose 5.5, KCl 5.4, MgSO_4_ 0.8; pH 7.4). The atria and great vessels were trimmed and discarded. The ventricles were cut into small pieces and incubated (37 °C, 20 min) repeatedly (5 to 6 times) in a buffer supplemented with collagenase type II (Worthington Biochemical Corporation, New Jersey, USA) and pancreatin (0.6 mg/mL; Gibco BRL). After each round of digestion, the supernatant was centrifuged (1200 rpm, 10 min), and the resulting cell pellet was resuspended in DMEM/M199 (4:1) supplemented, with 5 % horse serum (Biochrom, Berlin, Germany), 10 % fetal bovine serum (Gibco by Thermo Fischer Scientific, Karlsruhe, Germany), penicillin G (100 U/mL; Gibco by Thermo Fischer Scientific, Karlsruhe, Germany), and streptomycin (100 μg/mL; Gibco by Thermo Fischer Scientific, Karlsruhe, Germany). Then, cells were pooled and separated on a discontinuous Percoll (GE Healthcare Bio-Sciences AB, Uppsala, Sweden) gradient (top density = 1.059 g/ml, bottom density = 1.082 g/ml) by centrifugation. The middle band at the interface of the two Percoll layers was collected. Cell damage was estimated based on lactate dehydrogenase activity released from damaged cells into the cell culture, using a colorimetric lactate dehydrogenase-Cytotoxicity Assay kit (BioVision, CA, USA). The absorbance was measured at 490–500 nm using a plate reader (Thermo Labsystems Multiskan Ascent, MD, USA), and the percentage of cytotoxicity was then calculated.

#### Experimental groups

To investigate the effects of Zn(ASA)_2_ upon H_2_O_2_-induced oxidative stress in cardiomyocytes, the cultured neonatal cardiomyocytes were divided into two groups: (1) vehicle + H_2_O_2_ group (n = 25 wells): cardiac cells were pre-treated with the vehicle (DMSO, 2 h), then incubated with H_2_O_2_; (2) Zn(ASA)_2_ + H_2_O_2_ group (n = 25 wells): cells were pre-treated with Zn(ASA)_2_ (10^−3^ M; 2 h), then incubated with H_2_O_2_. The concentration of H_2_O_2_ was 500 µM, the incubation time was 2 h.

### Statistics

All data is expressed as mean ± standard error of the mean (SEM). Intergroup comparisons were performed by using one-way analysis of variance, followed by a Student’s unpaired t test with Bonferroni’s correction for multiple comparisons. A value of P <0.05 was considered statistically significant.

## Results

### Biochemical parameters

**Fig. 2:**
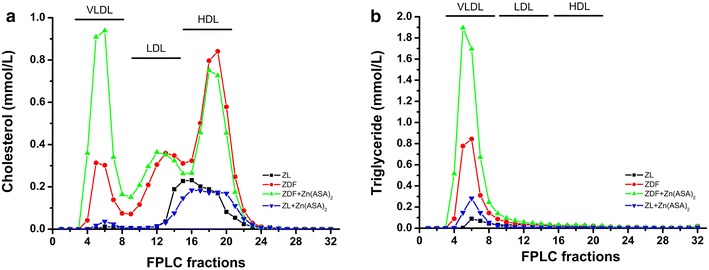
Total cholesterol and triglyceride levels of fast performance liquid chromatography (FPLC) fractions. The *graph* shows **a** cholesterol and **b** triglyceride concentrations of FPLC fractions from pooled serum. *Zn(ASA)*_*2*_ indicates a zinc complex of acetylsalicylic acid, *ZDF* Zucker diabetic fatty rats, *ZL* Zucker lean, *VLDL* very low density lipoprotein, *LDL* low density lipoprotein, *HDL* high density lipoprotein

The plasma glucose levels were significantly higher in ZDF rats compared to ZL animals (49.4 ± 2.8 mM vs. 11.8 ± 0.7 mM, P < 0.05), indicating the manifestation of an overt diabetes. When ZDF rats received daily oral administration of Zn(ASA)_2_ for 24 days, the plasma glucose levels were significantly reduced in comparison to ZDF rats (Table 1). Total cholesterol, HDL cholesterol, and free non-esterified fatty acid levels were significantly higher in ZDF rats than in ZL rats, and the Zn(ASA)_2_ treatment provided no changes. Additionally, triglyceride levels in the diabetic group were significantly elevated compared to both ZL groups. Treatment with Zn(ASA)_2_ further significantly increased plasma triglyceride concentrations in the diabetic rats, while having no effect upon the lean group. As the triglycerides were abnormally high, the LDL cholesterol calculation could not be performed. Therefore, the serum lipoproteins were separated by fast performance liquid chromatography (FPLC). Three major peaks were detected, representing the lipoprotein classes VLDL, LDL, and HDL, respectively (Fig. 2). Peak size analysis confirmed increased levels of HDL (Table 1; Fig. 2) and showed high VLDL and LDL (Fig. 2) in diabetic rats. The administration of Zn(ASA)_2_ had further increased VLDL levels, while there was no effect on LDL cholesterol. Furthermore, serum insulin levels were similar among the groups with a tendency toward increase in the nondiabetic control group treated with Zn(ASA)_2_. Plasma GOT/AST concentrations, which show the degree of hepatic disturbance, were also identical to those of the untreated rats.

**Table 1:** Biochemical parameters

Parameters	ZL (n = 5–9)	ZDF (n = 7)	ZDF + Zn(ASA)_2_ (n = 5–6)	ZL + Zn(ASA)_2_ (n = 6–10)
Plasma glucose (mmol/L)	11.8 ± 0.7	49.4 ± 2.8*^$^	39.0 ± 3.6*^#$^	11.4 ± 0.7
Plasma HDL cholesterol (mmol/L)	0.27 ± 0.01	1.96 ± 0.11*^$^	1.83 ± 0.06*^$^	0.28 ± 0.02
Plasma total cholesterol (mmol/L)	2.56 ± 0.13	8.68 ± 0.89*^$^	9.27 ± 0.95*^$^	2.24 ± 0.14
Plasma triglycerides (mmol/L)	1.0 ± 0.2	13.5 ± 1.1*^$^	17.4 ± 1.3*^#$^	1.1 ± 0.1
Non-esterified fatty acid (mmol/L)	0.21 ± 0.04	0.45 ± 0.07*^$^	0.49 ± 0.09*^$^	0.22 ± 0.01
Serum insulin (μg/L)	0.75 ± 0.10	0.71 ± 0.08	0.70 ± 0.09	1.33 ± 0.25
Plasma GOT/AST (U/L)	64 ± 4	77 ± 12	107 ± 26	81 ± 4

Biochemical profile of ZDF (Zucker diabetic fatty) and Zucker Lean (nondiabetic) rats after 24 days of treatment with either vehicle or zinc complex of acetylsalicylic acid (Zn(ASA)_2_). Values are mean ± SEM. * P < 0.05 vs ZL, ^#^ P < 0.05 vs ZDF, ^$^ P < 0.05 vs ZL + Zn(ASA)_2_

*GPT* indicates glutamate–oxaloacetate transaminase, *AST* aspartate aminotransferase, *n* number of animals analysed

### General characteristics and LV wall thickness

During 24 days of treatment, the ZL rats gained (+0.20 ± 2.8 g), whereas the ZDF rats lost weight (−75 ± 38 g), without reaching statistical significance (P > 0.05). Treatment with Zn(ASA)_2_ had no effect on body weight loss (ZDF + Zn(ASA)_2_: −17 ± 6 g; ZL + Zn(ASA)_2_: −7 ± 2 g). Furthermore, the body weight, heart weight, heart-to-body weight ratio, and heart weight-to-tibia length ratio were similar among the groups (Table 2). LV wall thickness, assessed by echocardiography, did not differ among the groups (Table 2).

**Table 2:** Body weight, heart weight, heart-to-body weight ratio, heart weight-to-tibia length ratio and left-ventricular wall thickness assessed by echocardiography

	ZL (n = 7–10)	ZDF (n = 7–8)	ZDF + Zn(ASA)_2_ (n = 5–6)	ZL + Zn(ASA)_2_ (n = 8–10)
Weights				
Body weight (g)	419 ± 9	376 ± 18	394 ± 27	416 ± 6
Heart weight (g)	1.36 ± 0.03	1.22 ± 0.04	1.35 ± 0.14	1.32 ± 0.03
Heart-to-body weight (g/kg)	3.26 ± 0.05	3.26 ± 0.11	3.39 ± 0.10	3.19 ± 0.07
Heart weight-to-tibia length (g/cm)	0.32± 0.01	0.30 ± 0.01	0.33 ± 0.03	0.30 ± 0.01
Left-ventricular wall thickness				
LVPW, d (mm)	2.49 ± 0.31	2.95 ± 0.42	1.95 ± 0.17	1.94 ± 0.20
LVPW, s (mm)	3.20 ± 0.27	4.24 ± 0.40	3.16 ± 0.19	2.69 ± 0.25

Values are mean ± SEM. *ZL* indicates Zucker lean, *ZDF* Zucker diabetic fatty acid, *Zn(ASA)*_*2*_ zinc complex of acetylsalicylic acid, *LVPW, d*, thickness of the left ventricular posterior wall in diastole, *LVPW, s* thickness of the left ventricular posterior wall in systole, *n* number of animals analysed

### Zn(ASA)_2_ improves ECG patterns

**Fig. 3:**
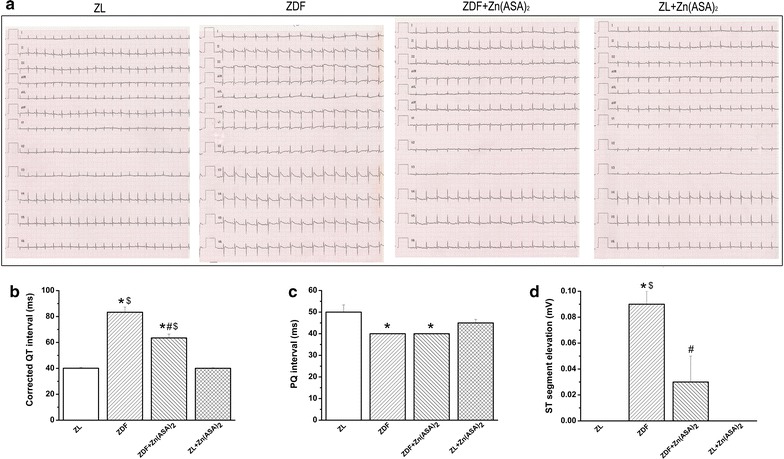
Zn(ASA)_2_ improves electrocardiographic pattern. **a** Representative surface 12-lead ECG tracing; **b** corrected QT interval; **c** PQ-interval, and **d** ST-segment elevation. Values are mean ± SEM, n = 6-10. *P < 0.05 vs ZL, ^#^P < 0.05 vs ZDF, ^$^P < 0.05 vs Zn(ASA)_2_, nQTc = QT/(RR/f)^1/2^. *Zn(ASA)*_*2*_ indicates a zinc complex of acetylsalicylic acid, *ZDF* Zucker diabetic fatty rats, *ZL* Zucker lean, *RR* corresponding to cardiac cycle length and f to frequency, *nQTc* QT intervals corrected with RR intervals

In ECG recordings, type-2 DM was associated with an elongated corrected QT-interval for heart rate, reduced PQ-interval, and moderate ST-segment elevation compared to ZL rats. Treatment with Zn(ASA)_2_ significantly reduced the prolonged corrected QT-interval and ST-segment elevation (Fig. 3).

### Zn (ASA)_2_ improves LV cardiac function

#### Basic hemodynamic data

The heart rate, systolic and diastolic blood pressures, mean arterial pressure, and the cardiac output were similar among all the experimental groups (Table 3).

**Table 3:** Hemodynamic parameters

Parameters	ZL (n = 7–10)	ZDF (n = 7)	ZDF + Zn(ASA)_2_ (n = 6)	ZL + Zn(ASA)_2_ (n = 9)
Basic hemodynamic data				
Heart rate (beats/min)	366 ± 12	346 ± 14	355 ± 12	373 ± 13
SBP (mmHg)	138 ± 5	151 ± 6	153 ± 6	141 ± 7
DBP (mmHg)	106 ± 3	108 ± 7	110 ± 5	107 ± 6
MAP (mmHg)	112 ± 4	122 ± 7	124 ± 5	118 ± 6
CO (ml/min)	31.0 ± 2.8	35.6 ± 5.3	35.6 ± 2.3	30.2 ± 4.1
Systolic function				
Stroke volume (µl)	85 ± 7	93 ± 13	100 ± 5	81 ± 11
Stroke work (mmHg*ml)	8.56 ± 0.88	10.71 ± 1.28	11.25 ± 0.78	7.86 ± 1.09
LVESP (mmHg)	131 ± 5	131 ± 6	128 ± 7	133 ± 7
E_es_ (mmHg/µl)	1.13 ± 0.13	0.75 ± 0.11	0.85 ± 0.06	1.18 ± 0.24
Diastolic function				
Tau (Weiss) (ms)	9.8 ± 0.3	10.3 ± 0.7	10.2 ± 0.3	9.9 ± 0.4

In vivo cardiac catheterization findings of ZDF (Zucker diabetic fatty) and Zucker lean (nondiabetic) rats after 24 days of treatment with either vehicle or Zn(ASA)_2_. Values are mean ± SEM, * P < 0.05 vs. ZL, ^$^ P < 0.05 vs. ZL + Zn(ASA)_2_

*Zn(ASA)*_*2*_ indicates a zinc complex of acetylsalicylic acid, *SBP* systolic blood pressure, *DBP* diastolic blood pressure, *MAP* mean blood pressure, *CO* cardiac output, *LVESP* left ventricular end systolic pressure, *E*_*es*_ the slope E_es_ of the left ventricular end-systolic pressure–volume relationship, *Tau* time constant of left ventricular decay, *n* number of animals analysed

#### Systolic function

**Fig. 4:**
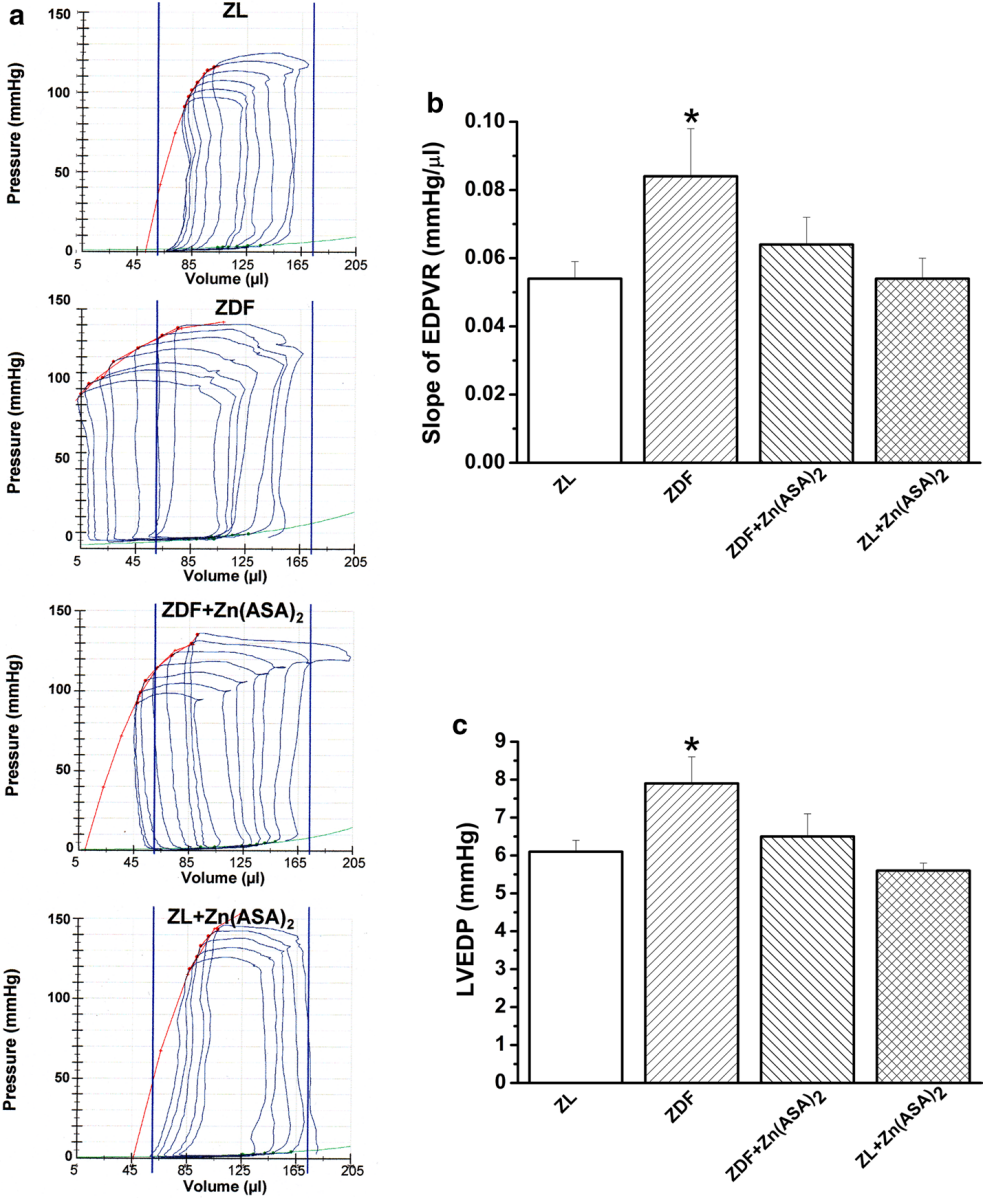
Zn(ASA)_2_ improves left-ventricular cardiac function. **a** Representative original pressure–volume loops registered during transient occlusion of the inferior vena cava. The slope of end-systolic pressure–volume relationship (*red line*) and end-diastolic pressure–volume relationship (EDPVR, *green line*); **b** the slope of end-diastolic pressure–volume relationship (EDPVR); and **c** left-ventricular end-diastolic pressure (LVEDP). Values are mean ± SEM, n = 6–10. *P < 0.05 vs ZL, ^#^P < 0.05 vs ZDF, ^$^P < 0.05 vs Zn(ASA)_2_. *Zn(ASA)*_*2*_ indicates a zinc complex of acetylsalicylic acid, *ZDF* Zucker diabetic fatty rats, *ZL* Zucker lean

The stroke volume, stroke work, and LVESP did not differ among the groups (Table 3). Figure 4 shows representative original pressure–volume loops registered during transient occlusion of the inferior vena cava. The end-systolic pressure–volume relationship of the diabetic rats demonstrated only slightly decreased slope (E_es_) in comparison to that of the ZL animals (Table 3).

#### Diastolic function

Increased slope of EDPVR and the LVEDP in ZDF animals suggested a markedly increased LV diastolic stiffness (Fig. 4). Moreover, these passive diastolic function parameters were similar in ZL and ZDF + Zn(ASA)_2_ animals. The LV active relaxation as assessed by Tau-w did not differ among the groups (Table 3).

### Zn(ASA)_2_ decreases cardiomyocyte hypertrophy

**Fig. 5:**
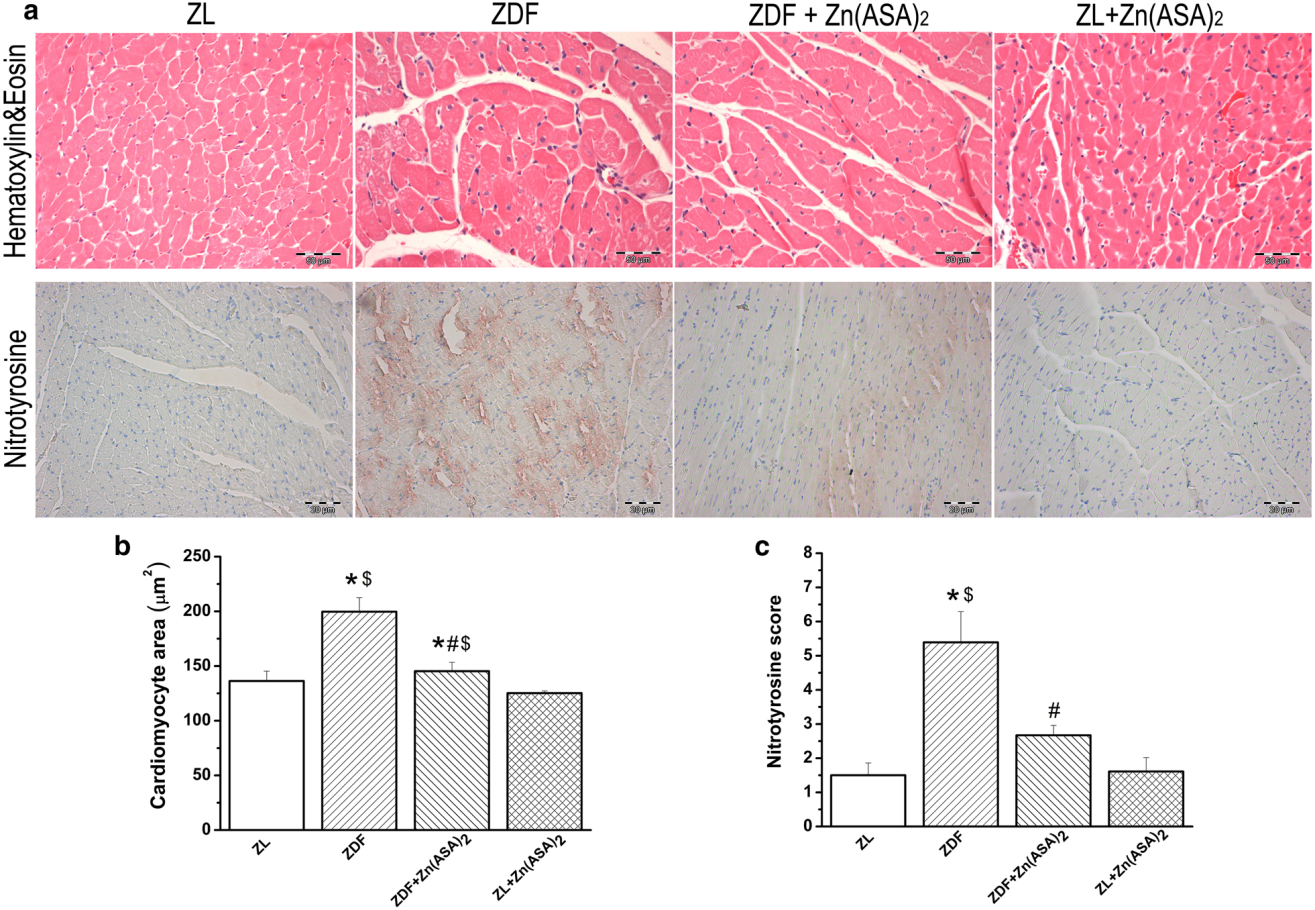
Zn(ASA)_2_ decreases cardiomyocyte hypertrophy and nitro-oxidative stress. **a** Hematoxylin and eosin staining micrographs of transverse sections of myocardium (magnification ×400; *scale bar*: 50 μm) and representative photomicrographs of nitrotyrosine immunohistochemistry staining; magnification ×200, *scale bar*: 20 µm. **b** Quantitative analysis of cardiomyocyte cross-sectional area using of ~20 cardiomyocytes in each group and **c** immunohistochemical scores for nitrotyrosine in the myocardium. Values are mean ± SEM, n = 6–9. *P < 0.05 vs ZL, ^#^P < 0.05 vs ZDF, ^$^P < 0.05 vs Zn(ASA)_2_. *Zn(ASA)*_*2*_ indicates a zinc complex of acetylsalicylic acid, *ZDF* Zucker diabetic fatty rats, *ZL* Zucker lean

Histological examination revealed that the cardiomyocyte transverse cross-section area in the ZDF rats was significantly increased in the H&E stained sections compared to the ZL animals (Fig. 5a, b). This enlargement was significantly decreased after the Zn(ASA)_2_ treatment.

### Zn(ASA)_2_ decreases nitro-oxidative stress

A significant increase in nitrotyrosine production could be observed in ZDF rats when compared with ZL animals. Treatment with Zn(ASA)_2_ significantly decreased nitrotyrosine production, as testified by decreased brown staining (Fig. 5a, c).

### Effect of Zn(ASA)_2_ on myocardial fibrosis

**Fig. 6:**
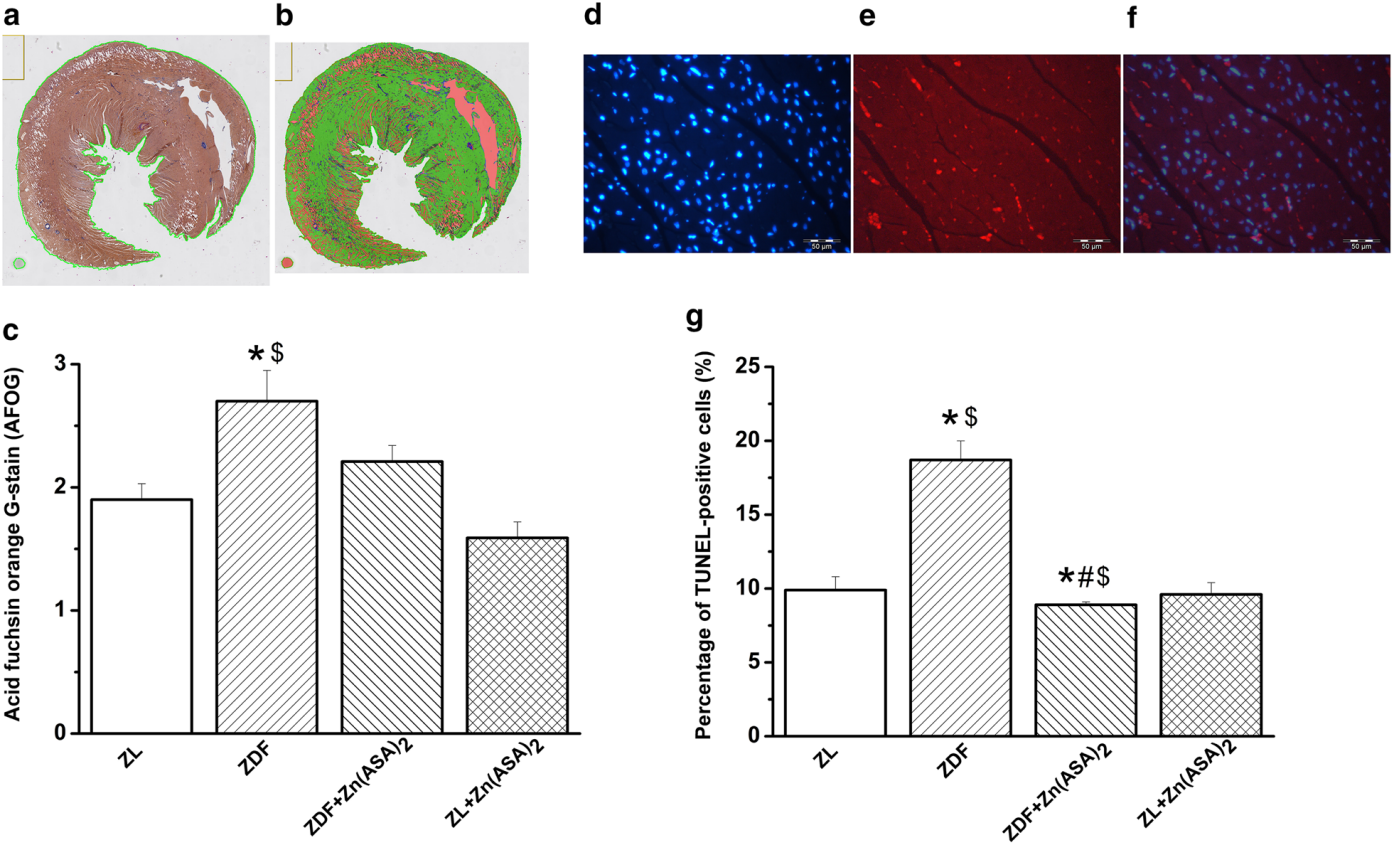
Effect of Zn(ASA)_2_ on myocardial fibrosis and DNA-strand breaks in cardiomyocytes. Representative photomicrographs showing the whole-slide after **a** the region of interest detection and **b** pixelwise classification of the tissue; *blue* fibrotic areas, *green* normal tissue, *orange* excluded areas in the acid fuchsin orange G (AFOG) stained sections. **c** Quantitative analysis of interstitial fibrosis in the myocardium. Representative photomicrographs of **d** nuclei with 4′,6-diamidino-2phenylindole (DAPI-stained nuclei, *blue*); **e** nuclei with fragmented DNA visualized by Terminal Deoxynucleotidyl Transferase-Mediated dUTP Nick End-Labeling (TUNEL) staining (TUNEL-positive nuclei, *red*), and **f** merged image (*red*/*blue* double stained) (magnification ×400, *scale bar*: 50 µm) from the same sample which belong to the ZDF group. **g** Quantification of TUNEL-positive cells for each group. Values are mean ± SEM, n = 6–10. *P < 0.05 vs ZL, ^#^P < 0.05 vs ZDF, ^$^P < 0.05 vs Zn(ASA)_2_. *Zn(ASA)*_*2*_ indicates a zinc complex of acetylsalicylic acid, *ZDF* Zucker diabetic fatty rats, *ZL* Zucker lean

The histopathologic examination of myocardial tissue, using acid fuchsin orange G-stain (AFOG), revealed a significant increase in fibrotic formation in ZDF rats compared to ZL animals. ZDF rats treated with Zn (ASA)_2_ showed a collagen content comparable to the ZL groups (Fig. 6a–c).

### Zn(ASA)_2_ decreases DNA strand breaks

A significant increase in the density of TUNEL-positive cell nuclei was observed in the myocardium of ZDF rats compared to ZL animals, indicating DNA-fragmentation. Treatment with Zn(ASA)_2_ significantly decreased the percentage of myocardial cells with DNA damage (Fig. 6d–g).

### Zn(ASA)_2_ regulates myocardial protein expression

**Fig. 7:**
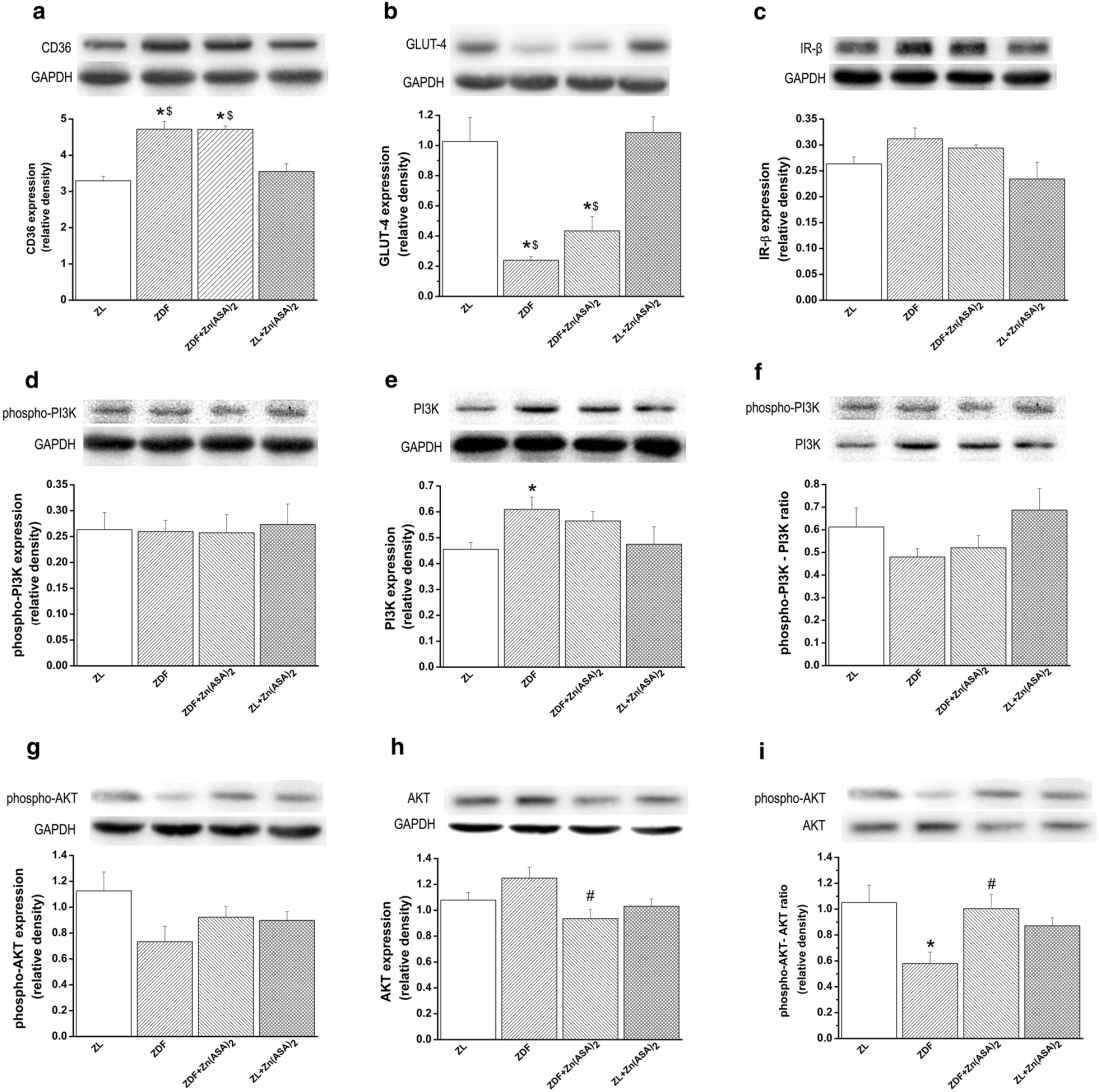
Effects of Zn(ASA)_2_ on myocardial protein expression. Immunoblot analysis for **a** CD36, **b** insulin receptor (IR)-β, **c** glucose transporter (GLUT)-4, **d** phosphorylated PI3K, **e** PI3K, **f** phosphorylated phosphatidylinositol 3-kinase-(PI3K)/PI3K ratio, **g** phosphorylated, **h** total AKT and **i** phosphorylated AKT/total Akt ratio protein band densities in the myocardium. Glyceraldehyde-3-phosphate dehydrogenase (GAPDH), housekeeping protein was used as reference. Values are mean ± SEM, n = 6–7. *P < 0.05 vs ZL, ^#^P < 0.05 vs ZDF, ^$^P < 0.05 vs Zn(ASA)_2_. *Zn(ASA)*_*2*_ indicates a zinc complex of acetylsalicylic acid, *ZDF* Zucker diabetic fatty rats, *ZL* Zucker lean

Western blot analysis (Fig. 7) revealed that the expression of CD36 was significantly increased, and GLUT-4 was decreased in ZDF rats compared to the nondiabetic group. The insulin receptor β expression was similar among the groups. Zn(ASA)_2_ had no effect on these proteins. Even though there was no significant change in phosphorylated-PI3K, type-2 DM promotes the expression of total PI3K compared to the control, but the ratio remained unchanged. Additionally, despite no significant alteration in phosphorylated and total AKT, ZDF rats showed a significant decrease in phosphorylated-AKT/AKT ratio compared to the ZL group. Treatment with Zn(ASA)_2_ significantly decreased total AKT and considerably increased the phosphorylated-AKT/AKT expression.

### Zn(ASA)_2_ reduces the cytotoxicity induced by H_2_O_2_ in cardiomyocytes

H_2_O_2_, at a concentration of 500 μM, induced 73 ± 2 % cytotoxicity in cultured neonatal cardiomyocytes, which was significantly decreased to 26 ± 1 % after a pre-treatment of cardiac cells with Zn(ASA)_2_.

## Discussion

The aim of this study was to investigate the effects of the oral administration of zinc and aspirin, in the form of bis(aspirinato)zinc(II) complex on diabetes-induced cardiac damage. Our results suggest that its blood glucose lowering effect with increased activation of the serine-threonine kinase AKT in the myocardium could, in part, explain the cardioprotective effects of Zn(ASA)_2_ upon DCM.

DCM has been defined as ventricular dysfunction along with abnormal myocardial structure in the absence of changes in blood pressure and coronary artery disease. Multiple pathophysiological mechanisms have been proposed to explain this phenomenon, such as myocardial fibrosis, myocyte hypertrophy, contractile dysfunction, changes in calcium handling and in mitochondrial function, abnormal cardiomyocyte loss by apoptosis and unsuccessful metabolic adaptation [30]. Despite all these mechanisms, hyperglycemia is considered to be one of the main pathogenic mechanisms of DCM. The potential role of a zinc complex of acetylsalicylic acid in treating DM has recently been demonstrated. An orally administered complex of zinc and aspirin ameliorated hyperglycemia and metabolic syndrome-like disorders in an animal model of type-2 DM [19]. The anti-diabetic effect of Zn(ASA)_2_ is also supported by our findings in ZDF rats, which showed that a treatment with Zn(ASA)_2_ caused a significant reduction of high plasma glucose levels. However, its administration did not have a significant impact on the lipid profile, except that the VLDL-triglyceride levels were increased further by the treatment. It might be that the Zn(ASA)_2_ lowers blood glucose levels by enhancing the conversion of excess glucose to triglycerides in the liver. However, we do not have unequivocal evidence at this point, and further investigations are needed to clarify these metabolic aspects of Zn(ASA)_2_ treatment. Its underlying mechanisms involve glucose uptake into the adipocytes by facilitating insulin’s binding to its receptor, leading to the activation of insulin receptor β-subunit, insulin receptor substrates, PI3K which in turn activates protein kinase B/AKT. Thus, the translocation of GLUT-4 to the adipocytes membrane stimulated by insulin, normalizes blood glucose levels in the experimental diabetic animals [31]. In the present study set up, Zn(ASA)_2_ does not influence insulin production however, the small amounts of insulin released by the pancreas can be more effective after a Zn(ASA)_2_ treatment. Furthermore, the insulin-mimetic activity of zinc complexes was previously examined in terms of free fatty acid release, and was found to inhibit free fatty acid release from isolated rat adipocytes treated with epinephrine (adrenaline) [31], also indirectly indicating their in vitro glucose-uptake ability into the cells.

Metabolic abnormalities present in diabetes (hyperinsulinemia, hyperlipidemia, and hyperglycemia) might be the initial event which causes endothelial dysfunction, inflammation, apoptosis, and oxidative stress [32]. In the present study, we demonstrated in an in vitro model, that Zn(ASA)_2_ protects cardiomyocytes from oxidative stress-induced damage. Furthermore, the presence of nitrotyrosine on proteins in the diabetic myocardium, as shown by our immunohistochemical staining, as a marker for nitro-oxidative stress in vivo, is in agreement with previous data [33, 34]. According to the semi-quantitative scoring of the staining, pronounced nitro-oxidative stress can be prevented by a Zn(ASA)_2_ treatment. Other studies supporting our observations have shown that zinc-induced overexpression of the antioxidant protein metallothionein protects the heart against diabetes-induced cardiac protein nitration in the development of DCM [35]. Zinc supplementation was sufficient to prevent DCM at least partially by reducing oxidative stress [36]. Molecular events of oxidative stress are lipid peroxydation [37], protein oxidation [38], and oxidative damage of DNA [39]. One of the most widely used methods for detecting DNA breaks and DNA fragmentation in tissue sections is TUNEL, which was described to detect cells undergoing apoptosis [40]. Oxidative stress-induced cell deaths, such as apoptosis, have important roles in the pathogenesis of DCM. In the present study, upon treatment of diabetic rats with Zn(ASA)_2_, the number of cardiac apoptotic nuclei was significantly decreased. In addition, apoptosis contributes to the loss of cardiac myocytes leading to adverse cardiac remodeling, including fibrosis and hypertrophy, impaired cardiac function, and eventually heart failure. The accumulation of extracellular matrix proteins, in particular collagens is one of the key processes in DCM. Sárközy et al. have shown an altered expression of several genes related to cardiac hypertrophy and remodeling [41]. Minerals, vitamins, and trace elements treatment in diabetic rats resulted in opposite gene expression changes, showing beneficial effects in DCM [41]. In the present study enhanced nitro-oxidative stress and apoptosis in the diabetic myocardium, and a high histopathological cardiac fibrosis score were shown in accordance with the literature, and were proven to be normalized by a Zn(ASA)_2_ treatment. Despite the ability of the antihyperglycemic agents, such as metformin and sulfonylureas, to maintain normoglycemia, many patients still develop DCM or are at risk for cardiovascular events [30]. To overcome these limitations and drawbacks for the use of conventional metabolic treatments, the findings of the present study may open novel therapeutic strategies, using Zn(ASA)_2_ directed against DCM.

Structural changes of the myocardium are often referred to as remodeling [42]. The main feature of this process is an increase in ventricular mass and at cellular level the hypertrophy of individual cardiomyocytes. An increase in myocyte profile surface area, as evident in the present work through enlarged cardiomyocytes in diabetic rats, has been observed. Potential causes of cardiomyocyte hypertrophy have been accredited to a number of factors, including mechanical stress [43], the sympathetic nervous system [44], the renin-angiotensin-aldosterone system [43], growth factors, and inflammatory cytokines [45]. Oral administration of Zn(ASA)_2_ protects the heart from pronounced hypertrophy of single cardiomyocytes. However, the LV wall thickness, assessed by echocardiography, did not significantly differ among the groups.

Metabolic disturbance in diabetes contribute to the development of DCM. Impaired cardiac function, due to cardiomyocyte hypertrophy, apoptosis, and fibrosis has been well documented in the setting of DCM [46]. Additionally, high glucose has been shown to exert detrimental effects on the myocardial contractile function in ZDF rats [47]. In the present study, with the use of in vivo cardiac catheterization and LV pressure–volume analysis, we showed that the slope E_es_ of ESPVR, as a load-insensitive index of contractility, was only slightly decreased in the diabetic rats. In consonance with our observations, our previous studies showed that LV contractility, with the use of load-independent variables, was not severely impaired in type-2 diabetic animals [24, 25]. Additionally, it has been shown that ZDF rats exhibited LV diastolic dysfunction [24], and that subclinical LV diastolic dysfunction is already present in the early phase of disturbed glucose metabolism, before the onset of type-2 DM, being mainly associated with the state of insulin resistance and not only with sustained hyperglycemia [48]. The administration of drugs that may reduce insulin resistance and related cardiovascular risk, would be useful in patients with type-2 DM at risk for developing DCM. In the present work, diabetes associated changes to the indexes of LV diastolic stiffness (LVEDP and slope of EDPVR) were significantly higher in type-2 diabetic rats, reflecting the impairment of passive, rather than active, LV diastolic function. The LV diastolic dysfunction was improved by Zn(ASA)_2_ treatment. Even though Xie and al. have shown only minor electrophysiological changes (in action potential duration and conduction velocity) during normoxic perfusion in 13–16 weeks old Zucker obese diabetic fatty rats [49], in the present study rats of the diabetic group presented a delayed repolarization of the ventricular myocardium manifested in prolonged QT intervals on the surface ECG, which was attenuated by a Zn(ASA)_2_ treatment. Our results showed that Zn(ASA)_2_ administration reduces the risk for ventricular tachyarrhythmia.

Being a key component of the insulin signaling pathway and its downstream targets, including serine/threonine protein kinase AKT, mammalian target of rapamycin (mTOR), and glycogen synthase kinase (GSK), PI3K plays important roles in cardiac adaptation including protein synthesis, inhibition of apoptosis, and fatty acid and glucose metabolism, as well as in gene expression and cell survival. In DM advanced glycation endproducts (AGEs)/receptor for AGEs interaction (RAGE) has been shown to contribute to the development of DCM [50]. A recent study indicates that AGEs trigger cardiomyocyte autophagy (a type II programmed cell death) by at least in part inhibiting the PI3K/AKT/mTOR pathway via RAGE [51]. The activation of the PI3K signaling increases serine phosphorylation of protein kinase B, and this results in the uptake of glucose by inducing the translocation of the GLUT-4 protein to the cell membrane. In the present study, the ratios of expression of phosphorylated AKT to total AKT, as measured by densitometry of Western blot, were significantly lower in heart samples from diabetic animals. The oral treatment of rats with Zn(ASA)_2_ reversed this alteration to the levels observed in ZL rats. It has been recently shown that zinc supplementation can rescue the detrimental effects of Akt2 gene deletion on the heart, probably associated with its insulin mimetic effect on the cardiac glucose-metabolism [52]. In the present study even though diabetes resulted in a reduction in myocardial GLUT-4 protein expression, a Zn(ASA)_2_ treatment had no effect upon this factor. The down-regulation of GLUT4 expression in the heart has been postulated to contribute to the altered substrate utilization and impaired contractility observed in diabetic cardiomyopathy [53]. Additionally, observations in GLUT4-deficient mice indirectly suggest that a down-regulation of GLUT4 may be involved in the pathogenesis of hypertrophy [54]. As our results showed that Zn(ASA)_2_ improves LV diabetic heart function not by decreasing myocardial hypertrophy but likely by its action on fibrosis, these observations may in part, explain why the Zn(ASA)_2_ treatment failed to regulate the expression of myocardial GLUT4 protein. All together, AKT activation seems to play a key role in the cardioprotective effects of Zn(ASA)_2_ against the DCM.

However some limitations of this study need to be addressed. ZDF rats represent a progressive and severe type-2 DM with diabetic complications that were left untreated until 25–26 weeks of age. We did not investigate whether improving metabolic control with a traditional oral antidiabetic treatment could also prevent diabetic cardiomyopathy. Additionally, the ZDF strain is an inbred rat model produced by a mutation in the leptin-receptor gene, whereas leptin deficiencies are not common in humans. However, ZDF rats were selected as an appropriate diabetic experimental model to verify the present study’s hypothesis.

## Conclusions

In summary, the oral administration of Zn(ASA)_2_ not only decreases blood glucose levels, but also alleviates type-2 diabetes-induced damage in rat cardiac tissue, in part via the activation of the myocardial AKT signalling pathway. We recently showed that Zn(ASA)_2_ protects the heart against myocardial ischemia in rats [22]. As a whole, our results suggest that Zn(ASA)_2_ might be an ideal therapeutic strategy for diabetic patients at risk for ischemic heart disease. Further detailed studies are required to reveal the exact mechanisms underlying Zn(ASA)_2_′s protective effects.

## Abbreviations

AFOG: acid fuchsin orange G
AST: aspartate aminotransferase
CO: cardiac output
DBP: diastolic blood pressure
DM: diabetes mellitus
EDPVR: slope of the end-diastolic pressure–volume relationship
E_es_: slope of the LV end-systolic pressure–volume relationship
f: frequency
ECG: electrocardiogram
FPLC: fast performance liquid chromatography
GAPDH: glyceraldehyde-3-phosphate dehydrogenase
GOT: glutamate-oxaloacetate transaminase
HDL: high density lipoprotein
H_2_O_2_: hydrogen peroxide
LDL: low density lipoprotein
LV: left-ventricular
LVEDP: left-ventricular end-diastolic pressure
LVESP: left-ventricular end-systolic pressure
MAP: mean arterial blood pressure
mTOR: mammalian target of rapamycin
RR: cardiac cycle length
SBP: systolic blood pressure
Tau: time constant of left ventricular pressure decay
TdT: terminal deoxynucleotidyl transferase
TUNEL: Terminal Deoxynucleotidyl Transferase-Mediated dUTP Nick End-Labeling
VLDL: very low density lipoprotein
ZDF: Zucker diabetic fatty
Zn(ASA)_2_: zinc complex of acetylsalicylic acid

## References

[1] Aneja A, Tang WH, Bansilal S, Garcia MJ, Farkouh ME (2008). Diabetic cardiomyopathy: insights into pathogenesis, diagnostic challenges, and therapeutic options. Am J Med.

[2] Regan TJ, Lyons MM, Ahmed SS, Levinson GE, Oldewurtel HA, Ahmad MR (1977). Evidence for cardiomyopathy in familial diabetes mellitus. J Clin Invest.

[3] Fein FS (1990). Diabetic cardiomyopathy. Diabetes Care.

[4] Schaffer JE (2003). Lipotoxicity: when tissues overeat. Curr Opin Lipidol.

[5] Unger RH, Orci L (2001). Diseases of liporegulation: new perspective on obesity and related disorders. Faseb J.

[6] van Herpen NA, Schrauwen-Hinderling VB (2008). Lipid accumulation in non-adipose tissue and lipotoxicity. Physiol Behav.

[7] Maharjan BR, Jha JC, Adhikari D, Vishwanath P, Baxi J, Alurkar VM (2008). A study of oxidative stress, antioxidant status and lipid profile in diabetic patient in the western region of Nepal. Kathmandu Univ Med J.

[8] Hotamisligil GS (2003). Inflammatory pathways and insulin action. Int J Obes Relat Metab Disord.

[9] Hundal RS, Petersen KF, Mayerson AB, Randhawa PS, Inzucchi S, Shoelson SE (2002). Mechanism by which high-dose aspirin improves glucose metabolism in type 2 diabetes. J Clin Invest.

[10] Yuan M, Konstantopoulos N, Lee J, Hansen L, Li ZW, Karin M (2001). Reversal of obesity- and diet-induced insulin resistance with salicylates or targeted disruption of Ikkbeta. Science.

[11] Chimienti F (2013). Zinc, pancreatic islet cell function and diabetes: new insights into an old story. Nutr Res Rev.

[12] Yoshikawa Y, Ueda E, Sakurai H, Kojima Y (2003). Anti-diabetes effect of Zn(II)/carnitine complex by oral administration. Chem Pharm Bull.

[13] Frommer DJ (1975). The healing of gastric ulcers by zinc sulphate. Med J Aust.

[14] Ozturk N, Olgar Y, Ozdemir S (2013). Trace elements in diabetic cardiomyopathy: an electrophysiological overview. World J Diabetes.

[15] Atahan E, Ergun Y, Belge Kurutas E, Cetinus E, Guney Ergun U (2007). Ischemia-reperfusion injury in rat skeletal muscle is attenuated by zinc aspartate. J Surg Res.

[16] Ozkan KU, Boran C, Kilinc M, Garipardic M, Kurutas EB (2004). The effect of zinc aspartate pretreatment on ischemia-reperfusion injury and early changes of blood and tissue antioxidant enzyme activities after unilateral testicular torsion-detorsion. J Pediatr Surg.

[17] Turut H, Kurutas EB, Bulbuloglu E, Yasim A, Ozkaya M, Onder A (2009). Zinc aspartate alleviates lung injury induced by intestinal ischemia-reperfusion in rats. J Surg Res.

[18] Singla AK, Wadhwa H (1994). zinc-aspirin complex—synthesis, physicochemical and biological evaluation. Int J Pharm.

[19] Yoshikawa Y, Adachi Y, Yasui H, Hattori M, Sakurai H (2011). Oral administration of Bis(aspirinato)zinc(II) complex ameliorates hyperglycemia and metabolic syndrome-like disorders in spontaneously diabetic KK-A(y) mice: structure-activity relationship on zinc-salicylate complexes. Chem Pharm Bull.

[20] Sarkozy M, Zvara A, Gyemant N, Fekete V, Kocsis GF, Pipis J (2013). Metabolic syndrome influences cardiac gene expression pattern at the transcript level in male ZDF rats. Cardiovasc Diabetol.

[21] Corsetti JP, Sparks JD, Peterson RG, Smith RL, Sparks CE (2000). Effect of dietary fat on the development of non-insulin dependent diabetes mellitus in obese Zucker diabetic fatty male and female rats. Atherosclerosis.

[22] Korkmaz S, Atmanli A, Li S, Radovits T, Hegedus P, Barnucz E (2015). Superiority of zinc complex of acetylsalicylic acid to acetylsalicylic acid in preventing postischemic myocardial dysfunction. Exp Biol Med.

[23] Hegedus P, Korkmaz S, Radovits T, Schmidt H, Li S, Yoshikawa Y (2014). Bis (Aspirinato) zinc (II) complex successfully inhibits carotid arterial neointima formation after balloon-injury in rats. Cardiovasc Drugs Ther.

[24] Radovits T, Korkmaz S, Loganathan S, Barnucz E, Bomicke T, Arif R (2009). Comparative investigation of the left ventricular pressure–volume relationship in rat models of type 1 and type 2 diabetes mellitus. Am J Physiol Heart Circ Physiol.

[25] Radovits T, Korkmaz S, Matyas C, Olah A, Nemeth BT, Pali S (2015). An altered pattern of myocardial histopathological and molecular changes underlies the different characteristics of type-1 and type-2 diabetic cardiac dysfunction. J Diabetes Res.

[26] Kmecova J, Klimas J (2010). Heart rate correction of the QT duration in rats. Eur J Pharmacol.

[27] Kass DA, Beyar R, Lankford E, Heard M, Maughan WL, Sagawa K (1989). Influence of contractile state on curvilinearity of in situ end-systolic pressure–volume relations. Circulation.

[28] Wang CW, Fennell D, Paul I, Savage K, Hamilton P (2011). Robust automated tumour segmentation on histological and immunohistochemical tissue images. PLoS One.

[29] Liaudet L, Soriano FG, Szabo E, Virag L, Mabley JG, Salzman AL (2000). Protection against hemorrhagic shock in mice genetically deficient in poly(ADP-ribose)polymerase. Proc Natl Acad Sci USA.

[30] Fuentes-Antras J, Picatoste B, Ramirez E, Egido J, Tunon J, Lorenzo O (2015). Targeting metabolic disturbance in the diabetic heart. Cardiovasc Diabetol.

[31] Yoshikawa Y, Ueda E, Kojima Y, Sakurai H (2004). The action mechanism of zinc(II) complexes with insulinomimetic activity in rat adipocytes. Life Sci.

[32] Tarquini R, Lazzeri C, Pala L, Rotella CM, Gensini GF (2011). The diabetic cardiomyopathy. Acta Diabetol.

[33] Kajstura J, Fiordaliso F, Andreoli AM, Li B, Chimenti S, Medow MS (2001). IGF-1 overexpression inhibits the development of diabetic cardiomyopathy and angiotensin II-mediated oxidative stress. Diabetes.

[34] Song D, Kuo KH, Yao R, Hutchings SR, Pang CC (2008). Inducible nitric oxide synthase depresses cardiac contractile function in Zucker diabetic fatty rats. Eur J Pharmacol.

[35] Cong W, Zhao T, Zhu Z, Huang B, Ma W, Wang Y (2014). Metallothionein prevents cardiac pathological changes in diabetes by modulating nitration and inactivation of cardiac ATP synthase. J Nutr Biochem.

[36] Lu Y, Liu Y, Li H, Wang X, Wu W, Gao L (2015). Effect and mechanisms of zinc supplementation in protecting against diabetic cardiomyopathy in a rat model of type 2 diabetes. Bosn J Basic Med Sci.

[37] Esterbauer H, Gebicki J, Puhl H, Jurgens G (1992). The role of lipid peroxidation and antioxidants in oxidative modification of LDL. Free Radic Biol Med.

[38] Sohal RS, Agarwal S, Dubey A, Orr WC (1993). Protein oxidative damage is associated with life expectancy of houseflies. Proc Natl Acad Sci USA.

[39] Imlay JA, Chin SM, Linn S (1988). Toxic DNA damage by hydrogen peroxide through the Fenton reaction in vivo and in vitro. Science.

[40] Loo DT (2011). In situ detection of apoptosis by the TUNEL assay: an overview of techniques. Methods Mol Biol.

[41] Sarkozy M, Szucs G, Pipicz M, Zvara A, Eder K, Fekete V (2015). The effect of a preparation of minerals, vitamins and trace elements on the cardiac gene expression pattern in male diabetic rats. Cardiovasc Diabetol.

[42] Cohn JN (1995). Structural changes in cardiovascular disease. Am J Cardiol.

[43] Yamazaki T, Yazaki Y (1999). Role of tissue angiotensin II in myocardial remodelling induced by mechanical stress. J Hum Hypertens.

[44] Sabbah HN (1999). The cellular and physiologic effects of beta blockers in heart failure. Clin Cardiol.

[45] Sano M, Fukuda K, Kodama H, Pan J, Saito M, Matsuzaki J (2000). Interleukin-6 family of cytokines mediate angiotensin II-induced cardiac hypertrophy in rodent cardiomyocytes. J Biol Chem.

[46] Huynh K, Bernardo BC, McMullen JR, Ritchie RH (2014). Diabetic cardiomyopathy: mechanisms and new treatment strategies targeting antioxidant signaling pathways. Pharmacol Ther.

[47] Bhatt NM, Aon MA, Tocchetti CG, Shen X, Dey S, Ramirez-Correa G (2015). Restoring redox balance enhances contractility in heart trabeculae from type 2 diabetic rats exposed to high glucose. Am J Physiol Heart Circ Physiol.

[48] Fontes-Carvalho R, Ladeiras-Lopes R, Bettencourt P, Leite-Moreira A, Azevedo A (2015). Diastolic dysfunction in the diabetic continuum: association with insulin resistance, metabolic syndrome and type 2 diabetes. Cardiovasc Diabetol.

[49] Xie C, Hu J, Motloch LJ, Karam BS, Akar FG (2015). The classically cardioprotective agent diazoxide elicits arrhythmias in type 2 diabetes mellitus. J Am Coll Cardiol.

[50] Yan SF, D’Agati V, Schmidt AM, Ramasamy R (2007). Receptor for advanced glycation endproducts (RAGE): a formidable force in the pathogenesis of the cardiovascular complications of diabetes and aging. Curr Mol Med.

[51] Hou X, Hu Z, Xu H, Xu J, Zhang S, Zhong Y (2014). Advanced glycation endproducts trigger autophagy in cadiomyocyte via RAGE/PI3K/AKT/mTOR pathway. Cardiovasc Diabetol.

[52] Sun W, Miao X, Zhou S, Zhang L, Epstein PN, Mellen N (2014). Zinc rescue of Akt2 gene deletion-linked murine cardiac dysfunction and pathological changes is metallothionein-dependent. J Mol Cell Cardiol.

[53] Garvey WT, Hardin D, Juhaszova M, Dominguez JH (1993). Effects of diabetes on myocardial glucose transport system in rats: implications for diabetic cardiomyopathy. Am J Physiol.

[54] Abel ED, Kaulbach HC, Tian R, Hopkins JC, Duffy J, Doetschman T (1999). Cardiac hypertrophy with preserved contractile function after selective deletion of GLUT4 from the heart. J Clin Invest.

